# Wearable Sensor Analysis of Movement Biomechanics and Lateralization in Dart Throwing

**DOI:** 10.3390/s25092862

**Published:** 2025-04-30

**Authors:** Anna Letournel, Joana Carvoeiro, João Elias, Daniel Lopes, Hugo Alexandre Ferreira

**Affiliations:** 1 Departamento de Ciências Biomédicas, Instituto Politécnico de Setúbal, Escola Superior de Saúde, Campus do Instituto Politécnico de Setúbal, Estefanilha, Edifício ESCE/ESS, 2914-504 Setúbal, Portugal; 2 Instituto de Biofísica e Engenharia Biomédica, Faculdade de Ciências da Universidade de Lisboa, 1749-016 Lisboa, Portugal; 3 Escola Superior de Tecnologia de Setúbal, Instituto Politécnico de Setúbal, Campus do Instituto Politécnico de Setúbal, Estefanilha, Edifício ESTS, 2914-504 Setúbal, Portugal

**Keywords:** dexterity, dart performance, handedness, real-time motion tracking, Inertial Measurement Units (IMU)

## Abstract

In darts, the dominant limb typically has an advantage due to its superior performance characteristics. However, with training, the non-dominant limb can achieve nearly similar accuracy. Research suggests that left-handed individuals tend to have more balanced dexterity between their hands compared to right-handed individuals, who show a stronger preference for their dominant hand. This may provide a slight advantage for left-handed players. This study analyzed 12 participants (male and female, aged 20–25 years), including one left-handed male and one left-handed female, with the rest being right-handed. Each participant completed 18 throws with both their dominant and non- dominant limbs. The data collection was conducted using the XSENS MVN Awinda motion capture system, which employs inertial sensors placed on the hand, forearm, upper arm, and shoulder of both limbs. The MT Manager software extracted values such as angular variation, acceleration, and angular velocity, ensuring precise and synchronized data for analysis. The results showed higher scores and shorter throw durations when using the dominant hand. The male participants scored higher with both the dominant and non-dominant limb. The left-handed female showed greater dexterity balance between both limbs and the left-handed male showed better coordination, supporting the idea that left-handed individuals may have a natural advantage in dexterity symmetry.

## 1. Introduction

Darts is a sport that transcends age and gender, requiring a high level of skill to score as many points as possible. While both arms are used in the sport, the dominant upper limb consistently outperforms the non-dominant limb in various aspects of motor performance [1]. This phenomenon is largely attributed to the greater neural and muscular development of the dominant hand, which is typically engaged in most daily tasks [1]. For example, when comparing the flexion–extension and pronation–supination movements of the elbow between the dominant and non-dominant limbs, the dominant limb tends to exhibit greater range and strength. This is due to the increased usage of the dominant limb in everyday activities that require fine motor skills such as writing, eating, and lifting objects [1].

Furthermore, research has shown that characteristics such as maximum voluntary contraction of the first dorsal interosseous muscle, grip strength, and pinch strength are consistently higher in the dominant arm. On average, these performance characteristics are found to be 3% greater in the dominant limb across various studies [2]. This finding challenges the earlier claims by Crosby and Wehbé [3], who suggested that the difference between the dominant and non-dominant limb was around 10% in these same characteristics. The discrepancy may arise from differences in methodology or the specific populations studied, but it is clear that the dominant limb consistently outperforms the non-dominant one, albeit with less pronounced differences than initially proposed.

One key factor that contributes to this disparity in performance is muscular development. The dominant limb is more frequently engaged in tasks that require fine motor control, thereby promoting greater muscle strength and coordination in the dominant limb [2]. Additionally, Miller et al. (2018) [4] demonstrated that individuals with an injured dominant hand often continue to favor their injured hand despite the non-dominant hand potentially having greater dexterity. This phenomenon suggests a strong psychological and functional reliance on the dominant hand, further reinforcing the idea that the dominance of one hand is not only a result of physical strength but also psychological conditioning and habitual use.

These findings provide valuable insight into the differences in motor performance between the dominant and non-dominant limbs, highlighting the complex interplay between muscle development, neural adaptation, and habitual use. This understanding is important in sports like darts, where precise and consistent motor performance is critical, and it underscores the importance of the dominant limb in achieving optimal performance.

In the context of throwing in dart games, it is therefore also predictable that the dominant limb will generally have an advantage [1]. However, dart throwing is a sport that can be trained to the point where the non-dominant limb can nearly match the performance of the dominant arm, although the dominant limb will still retain some advantage. This suggests that the motor skills required for dart throwing, including accuracy and consistency, can be developed in both limbs with sufficient practice [2].

This does not imply that practicing with the non-dominant limb from the outset will automatically provide an advantage over someone who practices with their dominant hand. The example of basketball shooting illustrates this point well—individuals who begin practicing with their non-dominant limb often have an advantage over those who start with the dominant limb. This phenomenon may be explained by the concept of brain lateralization, which refers to the division of labor between the left and right hemispheres of the brain. The dominant hand is typically controlled by the hemisphere that is more specialized for fine motor skills, spatial awareness, and task-specific actions. However, when the non-dominant hand is trained, the brain can develop new neural pathways, improving the dexterity and accuracy of the non-dominant hand [5].

Interestingly, studies have shown that training the non-dominant hand can lead to significant improvements in performance, although it may take longer to reach the same proficiency as the dominant hand. This suggests that the non-dominant hand can be trained to become highly functional in activities such as dart throwing, but it may never fully overcome the initial advantages provided by the dominant hand [4].

Furthermore, brain plasticity—the ability of the brain to reorganize itself by forming new neural connections—plays a crucial role in this process. Through consistent practice, the brain can rewire neural circuits to improve the motor capabilities of the non-dominant hand, albeit not to the extent of the dominant hand’s performance in highly skilled tasks [6]. In this sense, training with the non-dominant hand may help an individual achieve better symmetry between their limbs, but it will not necessarily result in an overall advantage, especially in a sport like darts, where precision and muscle memory play a significant role.

A study examining hand dominance and dexterity found that individuals with a dominant left hand exhibit greater symmetry in dexterity between their dominant and non-dominant hands compared to those with a dominant right hand. Specifically, left-handed individuals tend to develop motor skills and functional abilities in their non-dominant hand that are more similar to those in their dominant hand, leading to higher bilateral coordination. In contrast, right-handed individuals demonstrate a more pronounced disparity between the two hands, with the dominant right hand showing greater skill and performance capabilities [7].

Given this distinction in hand function, it can be expected that in a sport like darts, individuals with a left-dominant hand may have an advantage over right-handed individuals. This advantage would be reflected in their ability to perform more evenly with both hands. Left-handed individuals tend to exhibit better dexterity in their non-dominant hand, and this bilateral coordination could enhance their overall throwing technique, whether using their dominant or non-dominant hand. On the other hand, right-handed individuals are more likely to rely predominantly on their dominant hand, and the disparity between the two hands may limit the use of their non-dominant hand for dart throwing [7].

Additionally, the increased symmetry in dexterity among left-handed individuals may provide an advantage in sports requiring fine motor control and coordination, such as dart throwing, where the precision and accuracy of the throw are essential. This phenomenon is attributed to the necessity for left-handed individuals to adapt to a world predominantly designed for right-handed use, leading them to develop increased proficiency with their non-dominant hand. Research on hand grip strength asymmetry supports this finding, indicating that left-handed individuals often have more equivalent strength between hands, likely due to the frequent use of their non-dominant hand in daily activities [8]. Studies suggest that the increased ambidexterity observed in left-handed individuals allows for more adaptable movement patterns [8,9], which can be beneficial in tasks requiring both hands or in situations where one limb may be more fatigued or less efficient [9,10].

## 2. Materials and Methods

This study investigates the biomechanics and lateralization in dart throwing using Inertial Measurement Unit (IMU) sensors to analyze motor control during the throwing process, providing detailed data on movement dynamics, posture, and limb coordination. It is part of a highly comprehensive experiment focusing specifically on the biomechanical aspects of dart throwing while also incorporating ECG, EEG, and eye-tracking data.

### 2.1. Dart Game Target

**Figure 1:**
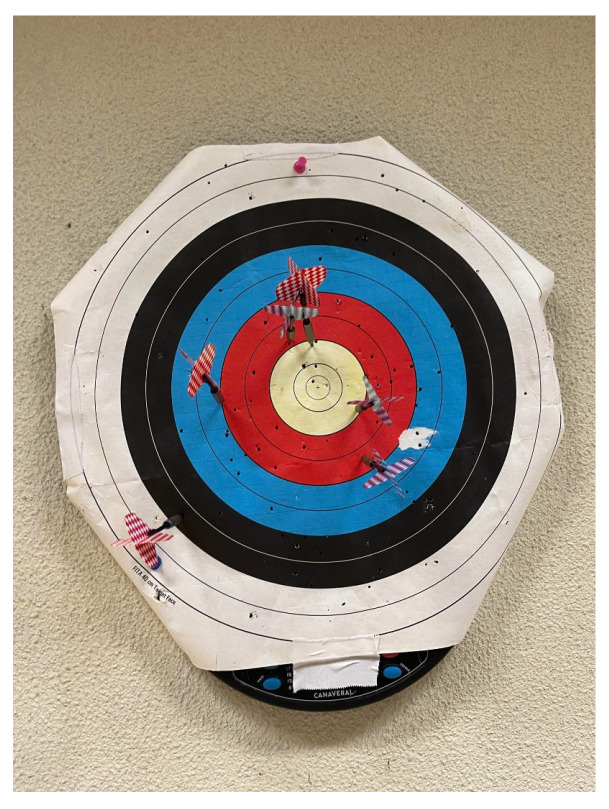
Archery target used in the experiment after a completed trial, with color-coded scoring zones clearly visible. The target’s scoring areas correspond to the participant’s performance, with the final score reflecting the accuracy and precision of the dart throws.

Participants performed the dart-throwing task under standardized conditions. The target used had a diameter of 40 cm and was positioned at a height of 1.73 m from the ground. Darts used in the task had an average weight of 10 g. The throwing distance was set at approximately 2.37 m from the participant, ensuring consistency across trials. In this study, an archery target paper with bright colors and consisting of 10 scoring zones in yellow, red, blue, black and white rings was used. The innermost yellow rings score ten and nine points, red rings score eight and seven, blue rings score six and five, black rings four and three, while the outermost white rings score two and one points, as can be seen in Figure 1. Each participant completed three trials per limb, with each trial consisting of six dart throws performed from a static position. This protocol was designed to evaluate motor performance and consistency under controlled conditions.

### 2.2. Participants

The study included 12 participants, 6 males and 6 females, aged between 20 and 25 years old. According to self-reported handedness, one male and one female participant were left-handed, while the remaining participants identified as right-handed. Each participant performed a total of 18 throws with each arm, both dominant and non-dominant, which were divided into three distinct trials per limb, with each trial consisting of six throws. Prior to the first movement, a two-minute rest period was recorded to establish a baseline measure. The objective of the task was to score as many points as possible per throw.

### 2.3. Motion Capture

**Figure 2:**
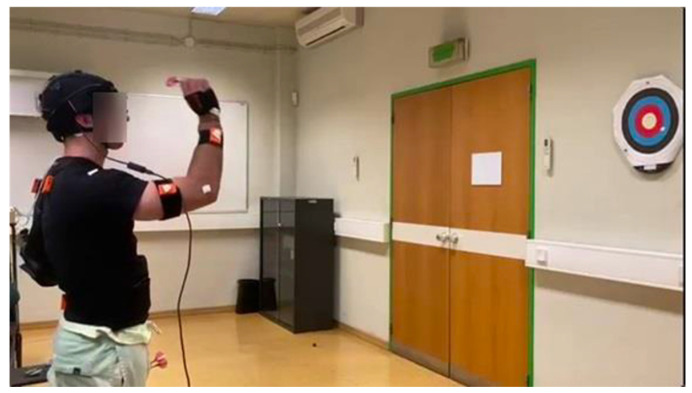
Experimental setup featuring the target and a participant equipped with wearable sensors during a trial. Sensors were placed on the participant’s hands, forearms, arms and shoulders of both limbs to capture biomechanical data, while the participant performed the dart-throwing task. This configuration allowed for real-time monitoring of movement patterns, posture, and throwing dynamics.

The XSENS MVN Awinda (Movella, Henderson, NV, USA) inertial motion capture system was employed to analyze the kinematics of dart throwing. The XSENS Movella products typically use a combination of IMUs, which consist of accelerometers, gyroscopes, and magnetometers, to capture precise movement data in three dimensions. The set-up can be seen in Figure 2 in which inertial sensors were strategically positioned on the hand, forearm, upper arm, and shoulder of both the dominant and non-dominant sides to capture detailed joint movement patterns.

**Figure 3:**
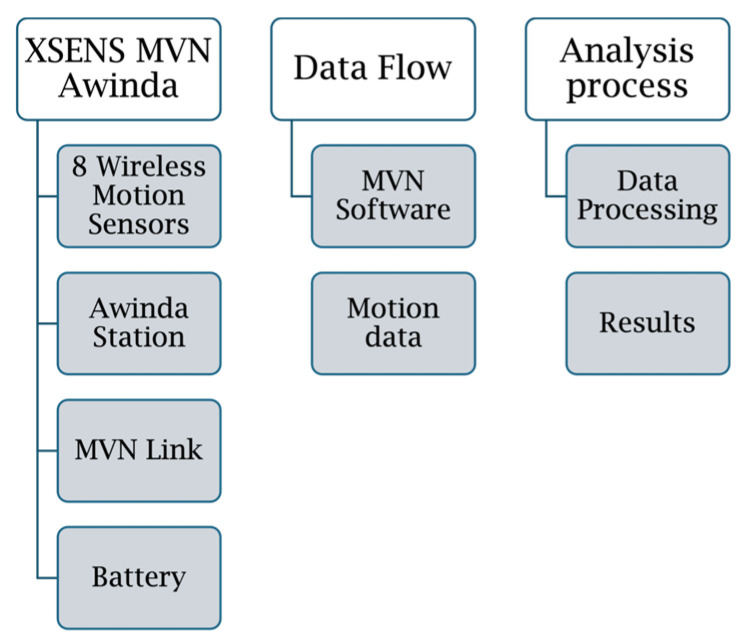
Block diagram illustrating the composition of the XSENS MVN Awinda motion capture system, the data flow, and the analysis process.

The data acquisition was performed using the MT Manager, version 2022.0.0 (Movella, Henderson, NV, USA) software, which enabled precise tracking and synchronization of motion data recorded at a sampling rate of 100 Hz. Figure 3 presents a block diagram illustrating the XSENS MVN Awinda motion capture system, its data flow, and the analysis process. The experiments were conducted within a maximum time span of 5 h to ensure optimal device performance, particularly in terms of battery life. The extracted kinematic parameters included joint angles, angular variation, acceleration, and angular velocity, providing a comprehensive assessment of movement dynamics. This methodological approach ensured high-resolution biomechanical analysis, facilitating an in-depth examination of motor lateralization and throwing mechanics. Importantly, this experimental setup allowed for the collection of data in a manner that closely resembles real-life dart throwing, as it did not impose movement restrictions on participants’ upper limbs.

However, participants were instructed to keep their lower limbs stationary throughout the experiment to minimize variations and ensure consistency across trials.

### 2.4. Detailed Data Analysis

The analysis examined the dart-throwing performance based on accuracy, consistency, and trial-based variations. The data were collected across all participants, evaluating key metrics such as target hit distribution and throwing precision. Throwing accuracy and consistency were analyzed to determine potential differences between limbs and across repeated trials.

The data collected from the inertial sensors were analyzed to assess the duration and performance of the throwing participant’s posture, focusing on how these factors correlated with the achieved score and the participant’s dominant side. The movements were analyzed in the transverse, sagittal, and frontal planes to capture rotational, forward–backward, and lateral adjustments during the throwing motion. The differences in movement execution between the limbs and across trials were examined to identify potential asymmetries and adaptations over time. Additionally, roll, pitch, yaw, gyroscopic data (X, Y, Z axes) were analyzed to examine angular velocity changes, while acceleration data (X, Y, Z axes) provided insights into movement dynamics, including variations in throwing force and stability. The postural assessment was conducted by analyzing the relative movement of the shoulders, as the sensors were only placed on the upper limbs. This evaluation provided insights into postural adaptations, asymmetries, and compensatory movements during the throwing motion.

**Figure 4:**
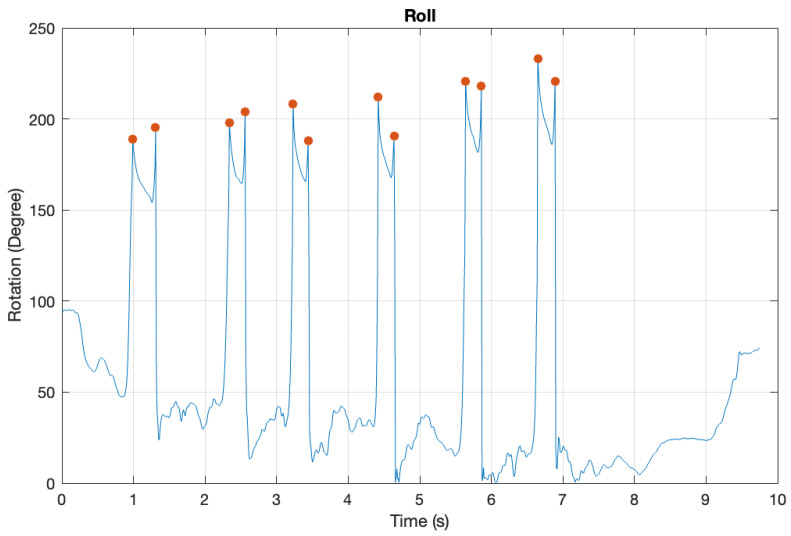
A detailed graphical analysis of the peak detection used to establish throwing time, incorporating the roll of the hand using MATLAB R2024b. The packet counter was converted to seconds based on the 100 Hz sampling frequency, ensuring uniform timing across all sensor data in the same collection.

Movement patterns from the hand sensor on the throwing side were examined to identify throwing intervals for all six throws in each trial. Distinct peaks in the transverse plane (roll) indicated the start and end of a throw, marked as red dots in Figure 4. These peaks defined the intervals comprising the throwing period.

Within these defined intervals, data from all relevant sensors were analyzed by computing the values for each trial. The extracted metrics included roll (rotation occurring in the transverse plane), pitch (movement within the sagittal plane), and yaw (rotation within the frontal plane), as well as gyroscopic and acceleration data along the three Cartesian axes (X, Y, and Z). For each participant for each trial, the median of the sensor-measured values during the throwing phase was calculated. To reduce the influence of outliers and ensure a more accurate representation of the performance, the average of these six median values was then computed to obtain a representative measure for the trial. Subsequently, the mean of the three trials was calculated to provide an overall performance measure for each participant for each limb. This approach facilitated a reliable analysis of the throwing performance by minimizing the impact of variability between individual attempts. The boxplots contrast the data for the left limb and right limb separately for throws executed with each limb. This approach allowed for a clear representation of variability, central tendency, and potential outliers, facilitating the comparison of limb-specific movement characteristics during the throwing task.

Postural stability was also assessed using boxplots, analyzing data from both shoulder sensors across all trials. This allowed for a comparative evaluation of shoulder movement stability in the transverse, sagittal, and frontal planes.

Coordination was assessed based on the stability of angular kinematics (roll, pitch, and yaw), the stability of relative movement between the shoulder and trunk, and the duration of the throw. The stability of roll, pitch, and yaw was evaluated using the standard deviation of the angular displacements recorded by the IMU, with lower variability indicating greater coordination. The relative movement between the shoulder and trunk was analyzed through positional deviations, where reduced displacement suggested improved postural control and upper limb coordination. The throw duration was measured across trials, with shorter and more consistent durations, reflecting efficient motor planning and execution.

## 3. Results

**Figure 5:**
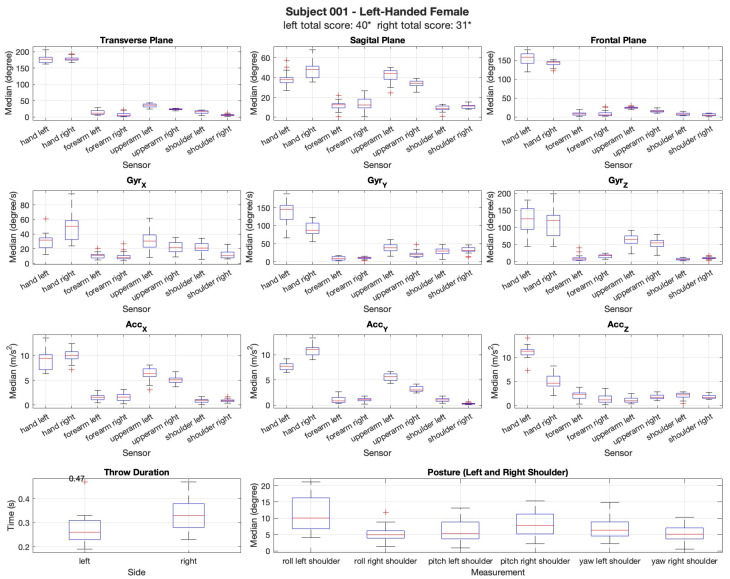
Data of the participant’s total score for both arms. Biomechanical data from a left-handed female participant were retrieved using XSENS and compared across both limbs. The analysis included measurements from the gyroscope along the (X, Y, Z axis), (*GyrX*, *GyrY*, *GyrZ*) measured in degrees/s and acceleration along the (X, Y, Z axis) (*AccX*, *AccY*, *AccZ*), measured in meters per second squared, which tracked movements in the transverse, sagittal, and frontal anatomical planes in degrees. Additionally, the throw duration was recorded for each limb, and posture was assessed for consistency in the upper body positioning throughout the dart-throwing task. The asterisk (*) indicates that a measurement reading failed for this participant, suggesting that the recorded final score may be an underestimation of the actual performance. The plus sign (+) represents the outliers.

**Figure 6:**
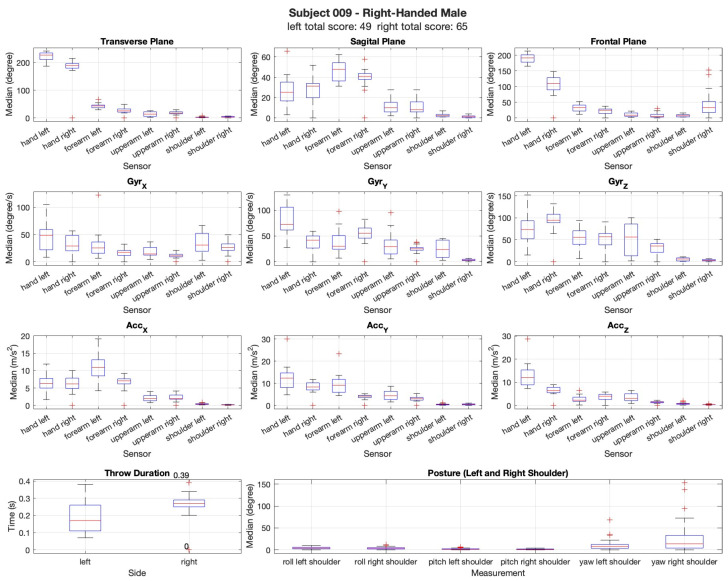
Data of the participant’s total score for both arms. Biomechanical data from a right- handed male participant were retrieved using XSENS and compared across both limbs. The analysis included measurements from the gyroscope along the (X, Y, Z axis), (*GyrX*, *GyrY*, *GyrZ*) measured in degrees/s and acceleration along the (X, Y, Z axis) (*AccX*, *AccY*, *AccZ*), measured in meters per second squared, which tracked movements in the transverse, sagittal, and frontal anatomical planes in degrees. Additionally, the throw duration was recorded for each limb, and posture was assessed for consistency in the upper body positioning throughout the dart-throwing task. The plus sign (+) represents the outliers.

The movements were analyzed in the transverse, sagittal, and frontal planes to capture rotational, forward–backward, and lateral adjustments during the throwing motion. The differences in movement execution between the limbs and across trials were examined to identify potential asymmetries and adaptations over time. Additionally, the gyroscopic data (X, Y, Z axes) were analyzed to examine angular velocity changes, while the acceleration data (X, Y, Z axes) provided insights into movement dynamics, including variations in the throwing force and stability. Postural assessment was conducted by analyzing the relative movement of the shoulders, as the sensors were only placed on the upper limbs. This evaluation provided insights into postural adaptations, asymmetries, and compensatory movements during the throwing motion. Regarding the results in the shoulder, small variations were recorded, as expected with the upper arm, forearm and hand, recording the highest variations among the participants as seen in Figure 5 and Figure 6. Rotation and flexion–extension movements recorded higher values during the dart throwing activity. Inversion and eversion exhibited higher values exclusively in the hand segment, whereas radial and ulnar deviation were present in both the dominant and non-dominant limbs. The female left-handed participant showed a higher similarity in dexterity with both the left and the right limb.

The results are shown in Figure 5 and Figure 6. For all the figures, the sex, hand dominance, total score with both hands, and movement biomechanics are presented. Although the results are shown with the left and right limbs side-by-side, it is important to note that they correspond to separate trials: throwing with the left hand and throwing with the right hand.

Figure 5 shows a left-handed female that scored 40 points with the left hand and 31 points with the right hand. The left-handed woman performed better with her left hand, as anticipated. It can be observed that the variation in movement between both limbs is similar. The variation in velocities recorded by the gyroscope for both limbs shows greater variation and higher nominal value in the hand, as expected, as well as for the acceleration. The other segments exhibit lower and less significant differences. In terms of throw duration, the left limb showed a shorter throw duration. In terms of posture, the analysis showed minimal variation in the upper body, suggesting a high level of consistency in the participants’ movements across the trials. This indicates that posture was maintained relatively stable throughout the experiment, with little fluctuation in the positioning of the limbs during the dart-throwing task.

Figure 6 shows a right-handed male that scored 49 points with the left hand and 65 points with the right hand. The right-handed male performed better with his right hand, as anticipated. This participant was selected because his score was similar to that of the female counterparts, as male participants generally scored higher than female participants overall during the experiment. In this case, the male participant exhibited less variability in movement between both limbs. The gyroscope-recorded velocities and acceleration data showed more consistent values for both limbs, indicating lower variability in the movement dynamics between the left and right limbs. In terms of throw duration, the right limb displayed a shorter throw variation. In terms of posture, the analysis showed greater variation in the upper body, indicating greater variability in the participant’s movements across the trials. This indicates that posture was less stable throughout the experiment, with more fluctuation in the positioning of the limbs during the dart-throwing task.

The left-handed male participant demonstrated the shortest throw times and achieved the highest scores, indicating a strong coordination between movement efficiency and accuracy. In terms of posture stability, he exhibited the lowest variation across the trials, suggesting a high degree of motor control and consistency in his throwing mechanics. The reduced variability in posture implies that the participant effectively minimized unnecessary movements, allowing for a more stable and repeatable throwing motion. This ability to maintain a controlled posture while executing rapid and precise movements may have contributed to his superior performance, reinforcing the link between movement efficiency and accuracy in dart throwing.

**Figure 7:**
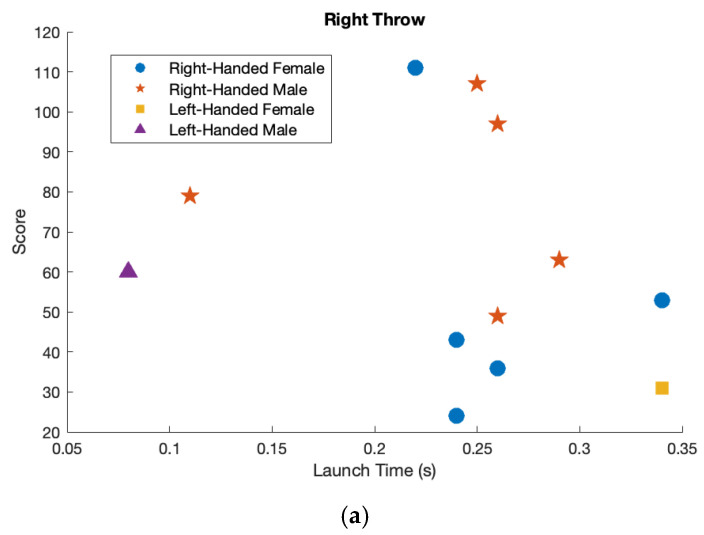

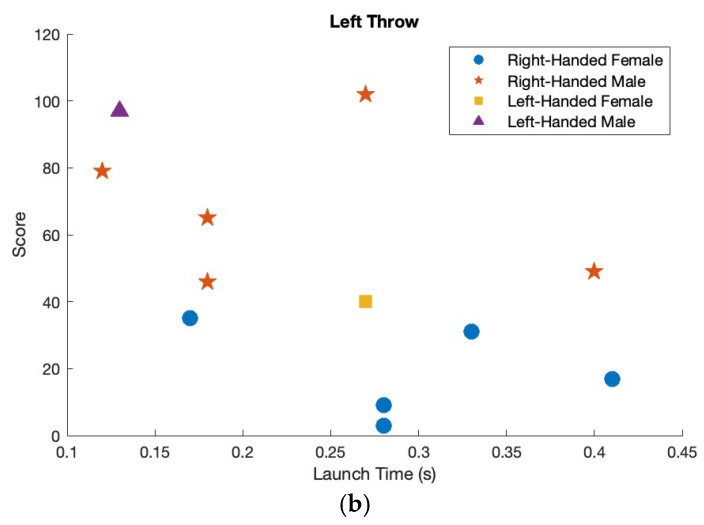
A graphical comparison of throw scores for all participants is shown, displaying performance with both the right and left hands. The right-handed females are represented by blue circles, left-handed female is represented by a yellow square, the right-handed males are represented by red stars and the left-handed male is represented by a purple triangle. (**a**) Right hand throw. (**b**) Left hand throw.

A comprehensive analysis was performed to investigate the relationship between the throw time and total score for all participants. This analysis aimed to identify any potential correlations between the duration of the dart-throwing motion and the accuracy of the throws, as measured by the total score achieved in the task. The results were examined across participants to determine whether faster throw times were associated with higher or lower scores as shown in Figure 7.

Overall, female participants scored lower than male participants with both the left and right hands. This trend was observed across all trials, with male participants consistently achieving higher scores regardless of which hand was used for the dart-throwing task. The difference in performance between sex was evident in both dominant and non-dominant hand throws. Throwing duration was slightly shorter for male participants compared to female participants. This difference was observed across both the left and right hands, indicating a marginally faster execution of the dart-throwing motion among the male participants. Considering the right-hand throw, both the left-handed male and female participants scored lower than the right-handed participants. However, the left-handed male was still the fastest, while the left-handed female exhibited the slowest throw duration. This highlights performance and timing differences between the left-handed and right-handed participants during the right-hand throws. Considering the left-hand throw, both the left-handed female and male participants scored higher with their left hand and exhibited a shorter throw duration. This suggests that, for both individuals, the left hand was not only more effective in terms of accuracy but also facilitated a faster execution of the dart-throwing motion.

Table 1, Table 2, Table 3 and Table 4 present the measured values of roll, pitch, and yaw for both limbs in all subjects. These tables provide the variability of these values in degrees, capturing the data for each subject’s dominant and non-dominant hand: Subject 001 is a left-handed female, while Subject 002 to 006 are right-handed females. Subject 007 is a left-handed male, and Subject 008 to 012 are right-handed males.

As anticipated, we observed a greater amplitude of movement in the hand compared to the shoulder. This is consistent with the nature of the task, where fine motor control of the hand is essential for the accurate release of the dart. The hand’s increased amplitude of movement reflects the intricate and dynamic adjustments needed during the final stage of the throw to ensure proper dart orientation and release trajectory.

In contrast, the shoulder exhibited the lowest variation in movement. This is expected as the shoulder primarily serves as the stabilizing joint, contributing to the overall control and positioning of the arm without the need for large or highly variable movements. The relatively low variation at the shoulder suggests that it functions more as a base of support, allowing for the more complex and variable movements of the forearm and wrist to occur with greater freedom. This observation underscores the shoulder’s role in providing stability and controlling the general movement path of the arm, while the hand fine-tunes the final throw dynamics.

Table 5 presents the average and standard deviation of the incremental velocity of the hand for all participants across each trial for both limbs.

The table reveals that drift and tremor are notably lower in the dominant hand for participants S001 and S007, both of whom are left-handed. Specifically, S001 demonstrates the smallest drift across both hands, with the values being particularly close for the right and left hands. This suggests a minimal difference in stability between the two limbs for this participant, indicating a potentially higher level of motor control or consistency in movement.

## 4. Discussion

In terms of overall performance, participants scored higher with their dominant hand, reinforcing the idea that the dominant limb typically performs better. This trend was evident across both the male and female participants, suggesting that handedness plays a significant role in task execution and accuracy.

The data showed greater coordination in the dominant limb for the majority of participants, which aligns with the expected pattern of superior motor control and fluidity in the dominant hand. This finding is consistent with the existing literature that suggests individuals typically exhibit better performance and more efficient movement patterns with their dominant limb [7].

Additionally, the duration and stability of the throw was shorter with the dominant limb, further supporting the notion of more efficient movement using the dominant limb [11]. This reduced throw time could reflect faster execution and a more refined motor pattern, as participants likely have greater familiarity and comfort with their dominant hand.

Interestingly, the left-handed female participant showed a lower degree of variation between both the left and right limbs. This suggests that, in this case, the non-dominant limb (right hand) exhibited performance characteristics more similar to the dominant limb (left hand), possibly indicating greater bilateral coordination or more balanced motor skills [12]. This finding contrasts with the typical pattern observed in right-handed individuals, where a distinction in performance between the two limbs is more pronounced.

The results indicate that the dominant limb performs better at dart throwing. These results contribute to our understanding of handedness and its influence on motor performance, highlighting both the expected trends and the exceptions in specific individuals. The novelty of this experiment lies in its comprehensive analysis of dart-throwing biomechanics using IMUs. Unlike previous studies that focus primarily on kinematics or dominant limb performance, this research examines coordination through roll, pitch, and yaw stability, relative shoulder and trunk movement, and throw duration. Additionally, it provides insights into handedness effects on performance, highlighting differences in motor control and consistency between dominant and non-dominant limbs. The study’s approach allows for a more ecologically valid assessment of throwing mechanics without restricting natural movement, contributing to a deeper understanding of upper limb coordination in precision sports.

In addition to the biomechanical factors observed, it is important to consider the degree of familiarity with the dart game, which may influence the results. The level of experience or familiarity with the game could affect the precision and variability of the movements, particularly in terms of the hand’s control over roll, pitch, and yaw. Experienced players are likely to demonstrate more consistent and refined motor patterns due to enhanced muscle memory and coordination developed over time. In contrast, less familiar or novice players may exhibit greater variability in their throwing mechanics as they rely more on trial and error to achieve the desired dart trajectory.

Thus, future studies should account for the participants’ level of experience with dart throwing, as this factor could influence the range of movement and the variability observed in the hand and shoulder. By incorporating the degree of familiarity with the game, a more comprehensive understanding of the biomechanics involved in dart throwing could be achieved, potentially leading to improved training methods and performance assessments.

However, it is crucial to recognize the limitations inherent to the study, which include a relatively reduced sample size. To further boost the research in this area, future studies should include larger groups of participants and diversity in laterality by including ambidextrous participants sorted using a laterality questionnaire. The incorporation of electroencephalography data, also recorded with sensor technology, introduces a promising avenue for probing the cognitive and neural processes underlying upper limb movements with playing darts to improve the understanding of this sport form.

## 5. Conclusions

In conclusion, the results of this study demonstrate that participants consistently performed better and completed the throw in a shorter duration when using their dominant limb. These findings highlight the inherent advantages provided by the dominant limb in tasks requiring motor precision, such as dart throwing. While the non-dominant hand showed potential for improvement through training, it was evident that the dominant hand maintained a performance edge. The data suggests that hand dominance plays a crucial role in motor control and performance, which can be attributed to factors such as neural coordination, muscle strength, and habitual usage. Further research is needed to explore the potential for training the non-dominant hand to reach performance levels closer to the dominant hand in such precision tasks. While this study focuses on dart throwing, the biomechanical methods—such as IMUs for measuring shoulder stability, velocity, acceleration, and timing—are applicable to various sports and daily activities requiring precise motor control. The kinematic principles observed here are relevant to upper limb movements in sports like tennis, baseball, and archery. These measures can also assess movement quality and optimize performance, providing real-time feedback for improved coordination, consistency, and injury prevention.

## Figures and Tables

**Table 1:** Mean and standard deviation of the variability of throw values in the hand for roll, pitch, and yaw (in degrees) for both limbs across all subjects.

Variable (Degrees)	Subject	Left Limb During Left Throw	Right Limb During Right Throw
Roll	Subject 001	177.055 ± 8.168	178.124 ± 5.485
Roll	Subject 002	175.773 ± 7.271	174.408 ± 9.212
Roll	Subject 003	163.921 ± 32.299	186.899 ± 16.601
Roll	Subject 004	174.448 ± 12.544	166.456 ± 15.662
Roll	Subject 005	171.099 ± 19.269	158.394 ± 13.121
Roll	Subject 006	91.564 ± 100.435	183.637 ± 4.212
Roll	Subject 007	174.439 ± 31.553	136.849 ± 53.371
Roll	Subject 008	206.759 ± 7.870	191.559 ± 16.322
Roll	Subject 009	222.507 ± 7.666	179.274 ± 32.170
Roll	Subject 010	195.715 ± 6.195	207.666 ± 11.051
Roll	Subject 011	194.164 ± 14.577	175.079 ± 19.277
Roll	Subject 012	190.461 ± 6.228	194.506 ± 14.261
Pitch	Subject 001	38.921 ± 7.241	48.675 ± 48.675
Pitch	Subject 002	39.354 ± 42.687	26.645 ± 4.948
Pitch	Subject 003	19.260 ± 8.177	24.427 ± 4.906
Pitch	Subject 004	21.099 ± 11.909	34.436 ± 11.509
Pitch	Subject 005	25.388 ± 9.022	45.697 ± 6.049
Pitch	Subject 006	11.003 ± 14.623	31.945 ± 14.990
Pitch	Subject 007	57.751 ± 16.230	35.626 ± 19.705
Pitch	Subject 008	18.855 ± 12.555	10.860 ± 4.937
Pitch	Subject 009	26.615 ± 11.857	27.927 ± 27.927
Pitch	Subject 010	43.386 ± 14.395	48.216 ± 10.566
Pitch	Subject 011	36.835 ± 9.054	28.047 ± 28.047
Pitch	Subject 012	38.800 ± 12.876	57.320 ± 8.286
Yaw	Subject 001	154.783 ± 17.768	141.610 ± 8.217
Yaw	Subject 002	148.863 ± 12.148	137.909 ± 5.300
Yaw	Subject 003	99.950 ± 21.276	147.477 ± 7.557
Yaw	Subject 004	135.481 ± 10.279	155.047 ± 5.707
Yaw	Subject 005	145.872 ± 20.501	128.751 ± 9.426
Yaw	Subject 006	65.879 ± 72.382	132.999 ± 4.776
Yaw	Subject 007	133.610 ± 23.298	126.528 ± 52.187
Yaw	Subject 008	112.598 ± 14.353	150.386 ± 10.317
Yaw	Subject 009	190.047 ± 10.405	106.184 ± 81.017
Yaw	Subject 010	142.490 ± 8.879	110.045 ± 9.000
Yaw	Subject 011	156.598 ± 6.509	149.870 ± 10.472
Yaw	Subject 012	136.547 ± 12.705	182.701 ± 6.682

**Table 2:** Mean and standard deviation of the variability of throw values in the forearm for roll, pitch, and yaw (in degrees) for both limbs across all subjects.

Variable (Degrees)	Subject	Left Limb During Left Throw	Right Limb During Right Throw
Roll	Subject 001	14.215 ± 8.140	7.278 ± 6.798
Roll	Subject 002	117.435 ± 16.389	92.356 ± 10.384
Roll	Subject 003	40.039 ± 16.436	50.772 ± 7.352
Roll	Subject 004	120.614 ± 22.474	109.882 ± 11.977
Roll	Subject 005	79.912 ± 21.501	42.070 ± 12.129
Roll	Subject 006	47.395 ± 60.792	108.028 ± 30.374
Roll	Subject 007	49.856 ± 19.095	83.004 ± 36.157
Roll	Subject 008	58.083 ± 9.383	62.540 ± 5.813
Roll	Subject 009	43.701 ± 9.263	27.356 ± 7.516
Roll	Subject 010	54.177 ± 27.930	112.685 ± 61.907
Roll	Subject 011	1.903 ± 0.977	2.935 ± 1.640
Roll	Subject 012	92.067 ± 14.173	85.620 ± 7.306
Pitch	Subject 001	12.010 ± 4.445	12.872 ± 7.384
Pitch	Subject 002	48.491 ± 12.840	55.649 ± 3.626
Pitch	Subject 003	33.802 ± 12.878	51.843 ± 2.555
Pitch	Subject 004	41.059 ± 12.406	36.990 ± 10.506
Pitch	Subject 005	50.082 ± 11.977	49.685 ± 4.010
Pitch	Subject 006	26.181 ± 30.323	72.913 ± 14.023
Pitch	Subject 007	74.354 ± 18.342	58.766 ± 27.687
Pitch	Subject 008	34.753 ± 10.137	28.669 ± 3.389
Pitch	Subject 009	46.352 ± 10.543	38.943 ± 10.353
Pitch	Subject 010	72.966 ± 7.143	70.902 ± 5.403
Pitch	Subject 011	2.302 ± 1.508	3.638 ± 2.061
Pitch	Subject 012	45.518 ± 8.539	55.088 ± 49.458
Yaw	Subject 001	8.209 ± 4.668	8.775 ± 7.864
Yaw	Subject 002	208.901 ± 14.477	62.950 ± 13.269
Yaw	Subject 003	76.780 ± 48.654	19.421 ± 3.439
Yaw	Subject 004	188.072 ± 32.602	74.115 ± 6.207
Yaw	Subject 005	165.808 ± 54.638	29.635 ± 14.354
Yaw	Subject 006	79.354 ± 99.590	114.562 ± 29.749
Yaw	Subject 007	57.927 ± 37.959	54.172 ± 23.778
Yaw	Subject 008	241.178 ± 43.424	53.600 ± 7.983
Yaw	Subject 009	31.624 ± 6.470	22.413 ± 7.965
Yaw	Subject 010	76.608 ± 52.949	101.783 ± 57.910
Yaw	Subject 011	2.420 ± 2.011	2.582 ± 2.408
Yaw	Subject 012	228.036 ± 37.658	56.077 ± 8.687

**Table 3:** Mean and standard deviation of the variability of throw values in the upper arm for roll, pitch, and yaw (in degrees) for both limbs across all subjects.

Variable (Degrees)	Subject	Left Limb During Left Throw	Right Limb During Right Throw
Roll	Subject 001	35.337 ± 5.272	23.367 ± 1.495
Roll	Subject 002	29.315 ± 4.855	13.644 ± 4.383
Roll	Subject 003	10.712 ± 2.693	50.939 ± 7.695
Roll	Subject 004	31.485 ± 12.429	33.389 ± 9.005
Roll	Subject 005	45.384 ± 19.026	29.925 ± 5.758
Roll	Subject 006	7.990 ± 10.737	10.098 ± 2.227
Roll	Subject 007	7.927 ± 3.012	2.702 ± 2.259
Roll	Subject 008	13.649 ± 5.384	16.554 ± 1.892
Roll	Subject 009	13.905 ± 5.751	17.547 ± 6.182
Roll	Subject 010	35.060 ± 12.283	7.558 ± 2.794
Roll	Subject 011	25.487 ± 5.643	27.061 ± 3.182
Roll	Subject 012	18.017 ± 4.082	21.011 ± 2.248
Pitch	Subject 001	41.602 ± 7.188	33.748 ± 3.048
Pitch	Subject 002	46.453 ± 7.379	33.831 ± 3.467
Pitch	Subject 003	21.942 ± 8.373	16.134 ± 3.581
Pitch	Subject 004	41.943 ± 8.065	41.936 ± 6.565
Pitch	Subject 005	29.216 ± 6.806	22.714 ± 4.201
Pitch	Subject 006	25.881 ± 29.192	56.906 ± 6.716
Pitch	Subject 007	47.325 ± 9.006	47.606 ± 21.010
Pitch	Subject 008	11.503 ± 6.813	12.928 ± 3.199
Pitch	Subject 009	11.305 ± 5.820	10.673 ± 5.742
Pitch	Subject 010	32.186 ± 4.029	33.750 ± 3.727
Pitch	Subject 011	21.141 ± 4.776	18.524 ± 3.678
Pitch	Subject 012	24.674 ± 6.635	36.636 ± 3.593
Yaw	Subject 001	24.172 ± 2.697	15.458 ± 3.688
Yaw	Subject 002	28.510 ± 4.431	7.882 ± 3.593
Yaw	Subject 003	9.146 ± 4.844	52.738 ± 16.416
Yaw	Subject 004	38.274 ± 14.566	23.671 ± 8.962
Yaw	Subject 005	40.884 ± 20.044	23.378 ± 6.761
Yaw	Subject 006	6.198 ± 8.302	14.669 ± 8.846
Yaw	Subject 007	20.340 ± 5.468	7.054 ± 5.429
Yaw	Subject 008	17.454 ± 9.026	14.200 ± 5.019
Yaw	Subject 009	10.125 ± 5.054	8.050 ± 5.704
Yaw	Subject 010	40.523 ± 13.570	10.085 ± 4.239
Yaw	Subject 011	26.306 ± 9.629	27.039 ± 5.565
Yaw	Subject 012	5.742 ± 2.287	13.180 ± 6.609

**Table 4:** Mean and standard deviation of the variability of throw values in the shoulder for roll, pitch, and yaw (in degrees) for both limbs across all subjects.

Variable (Degrees)	Subject	Left Limb During Left Throw	Right Limb During Right Throw
Roll	Subject 001	14.456 ± 5.481	5.285 ± 2.770
Roll	Subject 002	24.576 ± 7.224	59.289 ± 19.796
Roll	Subject 003	39.871 ± 29.007	8.925 ± 2.541
Roll	Subject 004	20.169 ± 9.591	41.327 ± 6.837
Roll	Subject 005	8.828 ± 3.230	3.685 ± 2.009
Roll	Subject 006	1.893 ± 2.464	7.903 ± 2.612
Roll	Subject 007	13.144 ± 3.685	6.713 ± 3.305
Roll	Subject 008	1.518 ± 0.902	7.127 ± 2.615
Roll	Subject 009	2.922 ± 1.557	3.767 ± 1.573
Roll	Subject 010	5.108 ± 3.783	13.892 ± 2.813
Roll	Subject 011	2.615 ± 1.679	3.447 ± 1.851
Roll	Subject 012	4.637 ± 2.466	1.903 ± 1.086
Pitch	Subject 001	8.799 ± 2.831	10.823 ± 1.965
Pitch	Subject 002	3.476 ± 1.980	5.546 ± 1.427
Pitch	Subject 003	2.958 ± 1.243	3.340 ± 1.099
Pitch	Subject 004	6.237 ± 2.023	5.065 ± 1.400
Pitch	Subject 005	7.101 ± 3.023	5.578 ± 1.739
Pitch	Subject 006	1.679 ± 1.881	3.700 ± 0.733
Pitch	Subject 007	1.232 ± 0.560	3.459 ± 1.888
Pitch	Subject 008	2.019 ± 1.028	3.949 ± 0.925
Pitch	Subject 009	2.775 ± 1.485	1.247 ± 1.002
Pitch	Subject 010	4.645 ± 1.457	4.862 ± 0.934
Pitch	Subject 011	1.463 ± 0.653	1.128 ± 0.453
Pitch	Subject 012	5.311 ± 1.390	4.486 ± 1.324
Yaw	Subject 001	8.023 ± 3.685	5.436 ± 2.933
Yaw	Subject 002	20.486 ± 6.412	105.008 ± 24.240
Yaw	Subject 003	21.783 ± 13.958	117.896 ± 52.448
Yaw	Subject 004	22.061 ± 9.155	218.921 ± 60.299
Yaw	Subject 005	7.038 ± 3.277	2.955 ± 1.730
Yaw	Subject 006	2.572 ± 3.806	14.054 ± 3.749
Yaw	Subject 007	21.272 ± 5.575	10.771 ± 5.127
Yaw	Subject 008	3.482 ± 1.745	19.262 ± 4.000
Yaw	Subject 009	7.675 ± 3.636	44.549 ± 24.594
Yaw	Subject 010	6.326 ± 3.602	15.329 ± 11.013
Yaw	Subject 011	3.246 ± 2.638	2.643 ± 1.571
Yaw	Subject 012	2.448 ± 1.862	4.965 ± 2.014

**Table 5:** Average and standard deviation of the incremental velocity (m/s) of the hand for all participants across each trial for both limbs.

Subject	Left Limb During Left Throw	Right Limb During Right Throw
Subject 001	0.06 ± 0.050	0.06 ± 0.055
Subject 002	0.10 ± 0.155	0.02 ± 0.018
Subject 003	0.21 ± 0.232	0.07 ± 0.047
Subject 004	0.05 ± 0.070	0.04 ± 0.050
Subject 005	0.11 ± 0.068	0.21 ± 0.033
Subject 006	0.08 ± 0.097	0.06 ± 0.064
Subject 007	0.02 ± 0.041	0.01 ± 0.007
Subject 008	0.05 ± 0.086	0.02 ± 0.013
Subject 009	0.04 ± 0.072	0.03 ± 0.026
Subject 010	0.20 ± 0.212	0.08 ± 0.131
Subject 011	0.09 ± 0.098	0.04 ± 0.022
Subject 012	0.03 ± 0.033	0.01 ± 0.010

## Data Availability

Data supporting the reported results can be obtained by writing to anna.letournel@ess.ips.pt.
